# STUDENT-LED INTERGENERATIONAL PROGRAMS IN NURSING HOMES

**DOI:** 10.1093/geroni/igae098.1572

**Published:** 2024-12-31

**Authors:** Frances Hawes, Inessah Cernohous

**Affiliations:** University of Wisconsin Eau Claire, Eau Claire, Wisconsin, United States; University of Wisconsin Eau Claire, Eau Claire, Wisconsin, United States

## Abstract

Recognized for fostering meaningful interactions across age groups, intergenerational programs offer numerous benefits, promoting social connections, empathy, and mutual understanding. This study compiles data from 25 intergenerational programs conducted by undergraduate students during their residency at nursing homes from 2020 to 2024. The aim is to pinpoint best practices and challenges in implementing such programs. The findings provide comprehensive summaries of the projects, detailing site contexts, methodologies, and results. We evaluate the effectiveness of these programs in various areas, including enhancing care quality, reducing social isolation, and improving the psychological well-being of nursing home residents. Furthermore, our analysis yields a consolidated understanding of both the successes and challenges encountered in intergenerational initiatives during this period, offering valuable guidance for future program development and implementation.

